# Supplementary material for: Generation of Hematopoietic-Like Stem Cells from Adult Human Peripheral Blood Following Treatment with Platelet-Derived Mitochondria
Source: Int J Mol Sci. 2020 Jun 15;21(12):4249. doi: 10.3390/ijms21124249 (PMC7352808; doi:10.3390/ijms21124249)
Supplement: Supplementary file 1 [file ijms-21-04249-s001.pdf]

Supplemental Figure Legend:

Figure S1. Scheme of the animal protocol for *in vivo* multiple differentiation of miCD34$^+$ HSC after transplantation into irradiated NSG mice. The purified miCD34$^+$ HSC (3×10^5 cells/mouse in 300 μl physiological saline, i.v. injection via tail vein, n = 10) were transplanted into NSG mice exposed to 300 cGy irradiation using a RS-2000 x-ray irradiator. Mice injected with an equal volume of saline served as a control group (n = 6).

Figure S1

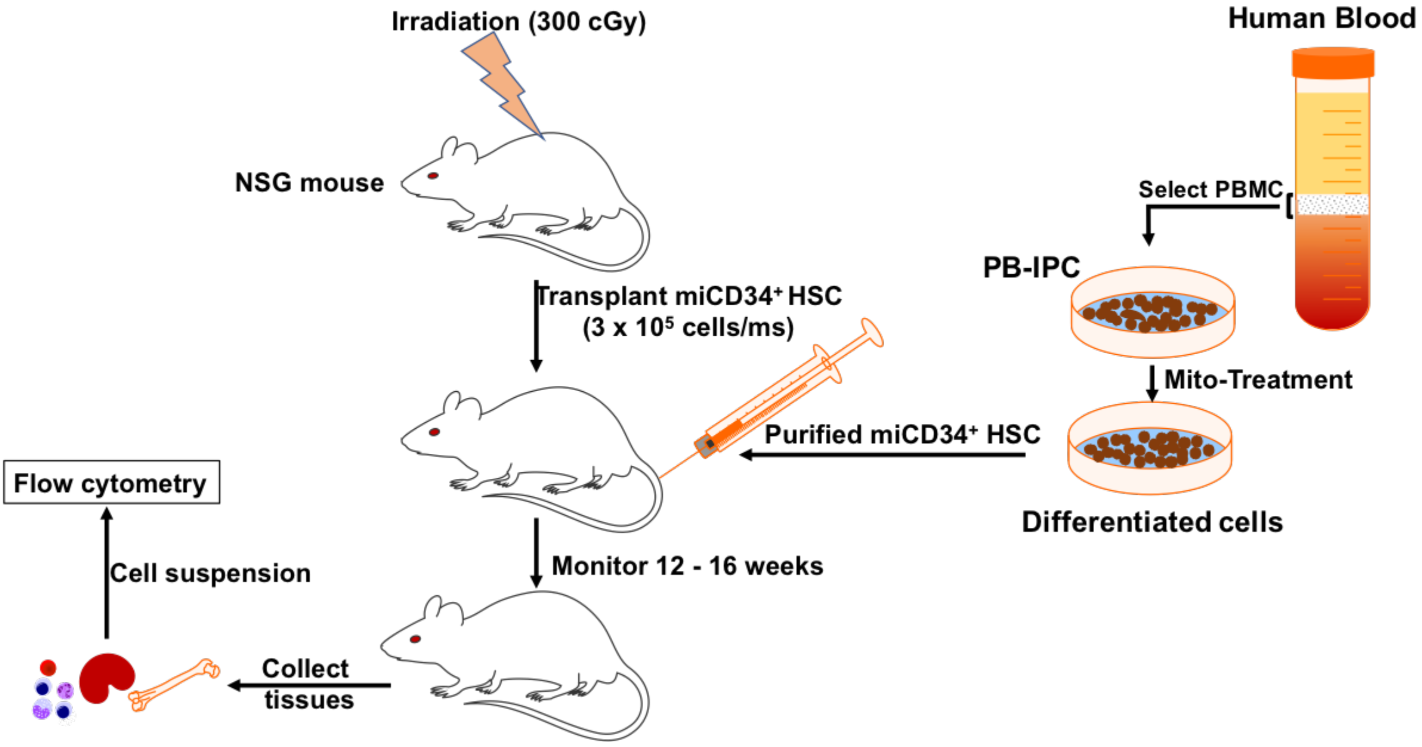

Figure S1. Scheme of the animal protocol for *in vivo* multiple differentiation of miCD34⁺ HSC after transplantation into irradiated NSG mice. The purified miCD34⁺ HSC (3×10^5 cells/mouse in 200 μl physiological saline, i.v. injection via tail vein, n = 10) were transplanted into NSG mice exposed to 300 cGy irradiation using a RS-2000 x-ray irradiator. Mice injected with an equal volume of saline served as a control group (n = 6).
